# From Doubt to Development: Professional Journeys of Novice CBT Therapists

**DOI:** 10.3390/bs15111504

**Published:** 2025-11-05

**Authors:** Aikaterini Tsamalidou, Panagiota Tragantzopoulou

**Affiliations:** 1 Department of Psychology, University of Derby, Derby DE22 3AW, UK; aika.tsamalidou@mc-class.gr; 2 School of Social Sciences, University of Westminster, London W1B 2UW, UK

**Keywords:** novice therapists, cognitive behavioral therapy, professional identity, supervision, emotional regulation

## Abstract

Novice therapists often experience a complex interplay of self-doubt, emotional strain, and professional uncertainty as they transition from training to independent clinical practice. This study explored the lived experiences of novice cognitive behavioral therapy (CBT) therapists, focusing on the challenges of early practice and the strategies employed to support regulation and growth. Seven early-career CBT therapists participated in semi-structured interviews, and data were analyzed using Interpretative Phenomenological Analysis (IPA). Two overarching themes were identified: professional identity challenges and self-beliefs, and strategies for emotional regulation and continuous development. Participants reported difficulties managing anxiety, boundary-setting, and integrating their professional and personal selves, particularly when working with complex presentations such as grief, self-harm, and personality disorders. At the same time, supervision, personal therapy, peer and family support, and ongoing professional development were seen as crucial in building resilience and sustaining competence. The findings suggest that training and professional structures should place greater emphasis on reflective practice, boundary management, and preparation for emotionally charged cases, while framing supervision as both a clinical and emotional resource. By highlighting the perspectives of novice therapists, the study underscores the importance of supportive systems in fostering resilience and sustainable professional growth.

## 1. Introduction

Cognitive Behavioral Therapy (CBT) is one of the most extensively researched and widely implemented psychological interventions (Knapp et al., 2015; David et al., 2018). Rooted in the cognitive model, CBT asserts that emotional distress and maladaptive behaviors are driven by distorted interpretations of events rather than the events themselves (Beck, 1995). These distortions often manifest as “automatic thoughts,” which are underpinned by deeper maladaptive core beliefs about the self, others, and the world.

CBT typically involves collaborative work between therapist and client to identify, evaluate, and modify unhelpful thinking patterns, alongside behavioral experiments and skills training. It is structured, goal-oriented, and time-limited, with a robust evidence base across a wide range of mental health conditions, including depression, anxiety disorders, obsessive-compulsive disorder (OCD), and post-traumatic stress disorder (PTSD) (Hofmann et al., 2012; Cuijpers et al., 2013). Adaptations such as Mindfulness-Based Cognitive Therapy (MBCT) and Dialectical Behavior Therapy (DBT) have expanded its scope to chronic depression and personality disorders (Gu et al., 2015).

While the method’s structured nature can be advantageous for clarity and efficiency, its effective delivery is highly dependent on the therapist’s skill, adaptability, and professional resilience. These aspects are often still developing in novice therapists, who face distinct challenges during their early years of practice.

### 1.1. Challenges Faced by Novice Therapists

Graduate training in psychotherapy can be an exciting period, yet it is also accompanied by significant challenges to both personal and professional development (Choudhury et al., 2019). Research shows that the first year of practice is a time of professional development and constant stress (Tryssenaar & Perkins, 2001). The transition from theory to practice often involves a steep learning curve, requiring therapists to navigate complex client presentations while managing their own emotional responses. In CBT specifically, the expectation to adhere to a structured, evidence-based model while remaining flexible to client needs can heighten performance pressure.

Novice therapists’ lack of experience may contribute to perfectionistic tendencies, which, when extreme, can cause stress and increase vulnerability to professional strain (D’Souza et al., 2011). This is compounded by the existential dimensions of the profession, as therapists deal with themes such as mortality, loneliness, and the pursuit of a meaningful career- elements that can shape their professional identity over the long term (Skovholt & Rønnestad, 2003). Early exposure to therapeutic training has also been shown to influence personal identity, sometimes triggering personal crises or disruptions to existing relationships (Råbu et al., 2016; Rath, 2008).

Emotionally, novice therapists frequently report feelings of responsibility, self-doubt, and uncertainty about whether their professional efforts are worthwhile (Råbu et al., 2016). Such challenges can become particularly acute when working with high-risk clients, including those with suicidal thoughts or those who ultimately take their own lives. Even experienced practitioners identify this as one of the most emotionally taxing aspects of the role (Råbu et al., 2016). For early-career therapists, such exposure can lead to immobilization, questioning of one’s effectiveness, and a significant drop in self-esteem (Thériault et al., 2009). These professional struggles frequently extend into therapists’ personal lives. For some, moments of self-perceived inadequacy within sessions trigger heightened anxiety, persistent rumination, and difficulties in mentally disengaging from work once at home (Thériault et al., 2009). Early-career therapists are particularly vulnerable to such pressures when progress with clients does not appear immediate or tangible. As Skovholt and Rønnestad (2003) argue, these “glamourized expectations” reflect the novice practitioner’s conviction that therapeutic effectiveness should be evident in rapid client improvement. When these expectations prove unrealistic, trainees often experience disillusionment, turning their doubts inward and questioning the adequacy of their professional skills. This erosion of confidence can undermine self-esteem and foster feelings of guilt, frustration, and professional inadequacy (Skovholt & Rønnestad, 2003). In more acute cases, such emotional strain has been found to interfere with the therapist’s capacity for empathy during sessions, thereby directly influencing the therapeutic relationship (Prikhidko & Swank, 2018; Stoltenberg & McNeill, 2009).

These difficulties echo findings from qualitative studies on therapists in various modalities. Demirtzidou and Tragantzopoulou’s (2025) work with novice integrative therapists revealed experiences of both physical and psychological strain, including fatigue, headaches, and countertransference reactions, often triggered by elevated caseloads, personal stressors, and feelings of inadequacy. Similarly, Tragantzopoulou et al. (2024) documented cognitive and emotional impacts such as decision-making difficulties, emotional numbing, and disconnection from clients, all of which can impair therapeutic alliance and effectiveness. While such experiences do not always develop into clinical burnout, they represent a spectrum of challenges that may predispose therapists to longer-term difficulties if unaddressed.

Other qualitative studies (Tragantzopoulou & Giannouli, 2023, 2020) have highlighted common emotional reactions among therapists, including bafflement, anxiety, and anger, which may also be influenced by personal biases. For novice CBT therapists, the interplay between structured treatment protocols and these emotional, personal, and professional demands makes the early career stage particularly complex. Qualitative evidence further illustrates this developmental trajectory. A grounded theory study with beginning CBT practitioners conceptualized their professional growth as a process of gaining professional confidence, progressing through three phases: reliance on external CBT methods, internalization of these methods, and eventually the emergence of self-confidence as therapists. The authors emphasized that self-reflection on personal qualities, values, and attitudes is central to this progression, suggesting that therapist development is not solely technical but deeply personal and reflective (Maruniakova & Rihacek, 2018).

### 1.2. Coping Strategies

Access to supportive resources is widely recognized as critical in enabling novice therapists to cope with the emotional and professional challenges of early practice. Beyond the strain of self-doubt and perceived incompetence, many beginning practitioners actively adopt coping strategies aimed at mitigating the negative impact of these experiences. Thériault et al. (2009), for instance, found that novice therapists often shift their attention away from self-criticism by prioritizing the therapeutic relationship, while also drawing on practices such as meditation, disclosing emotions to significant others, and seeking guidance and validation from supervisors. Supervision plays a central role, offering a safe space for reflection, skill development, and emotional processing. In a qualitative study, participants described supervision as instrumental in reducing anxiety, normalizing experiences, and sustaining a growth mindset (Demirtzidou & Tragantzopoulou, 2025). Recent qualitative evidence has also illuminated the processes that make supervision particularly effective for novice CBT practitioners. Effective supervision sessions were characterized by a collaborative exchange, supervisees’ active engagement with learning goals, and the supervisor’s ability to tailor guidance to individual needs within a supportive atmosphere. These relational and personalized dynamics have been linked to greater improvements in competence and confidence, underscoring the importance of flexible and empowering supervisory approaches in early professional development (Paunov et al., 2025). Personal therapy and peer support have also been identified as valuable, enabling therapists to address personal triggers and benefit from shared experiences (Tragantzopoulou et al., 2024). Organizational factors, such as manageable caseloads and a supportive work culture, further enhance resilience and professional development.

Although CBT is one of the most extensively researched therapeutic approaches (Hassan Kariri & Almubaddel, 2024), the literature has disproportionately focused on its effectiveness for clients rather than on the lived experiences of practitioners themselves. Where therapist perspectives have been explored, studies often concentrate on experienced practitioners or on counsellors working across eclectic or integrative modalities. This leaves a gap in understanding how early-career CBT therapists navigate the transition from training to independent practice within CBT’s structured and outcome-driven framework. Moreover, while the challenges faced by novice therapists have been documented in broad terms, there has been comparatively limited exploration of the coping strategies they employ to manage professional and emotional demands. While prior research has highlighted coping strategies broadly, there remains limited qualitative understanding of how novice CBT therapists implement these strategies in the context of structured CBT practice. By foregrounding the voices of early-career practitioners, this study contributes novel insights into the interplay between professional identity, emotional regulation, and practical coping mechanisms, demonstrating how these experiences shape ongoing development and therapist wellbeing. Thus, the present study seeks to fill a gap in the literature by examining the lived experiences of novice CBT therapists in the early stages of their careers. Specifically, it investigates how they navigate cognitive and emotional challenges, construct professional identity, and employ coping strategies to sustain personal and professional growth. This focus allows for concrete recommendations to enhance training, supervision, and support structures for early-career CBT practitioners.

## 2. Materials and Methods

### 2.1. Design

This study employed a qualitative research design, chosen for its capacity to provide an in-depth understanding of participants’ subjective experiences, attitudes, and beliefs within their social and cultural contexts. Qualitative methods are considered especially suitable for exploring complex human emotions and perceptions (Creswell, 2013; Denzin & Lincoln, 2011).

### 2.2. Participants

Seven participants were recruited, aligning with the recommended sample size for Interpretative Phenomenological Analysis (IPA), which typically ranges from six to eight (Turpin et al., 1997). All were novice CBT therapists with a maximum of five years’ experience in psychotherapy (see Table 1). Participants were aged 18 years or older and were exclusively Greek, ensuring a shared cultural context. All interviews were conducted in Greek, the participants’ native language, to facilitate rich and nuanced descriptions of their experiences. The inclusion of exclusively Greek participants also ensured cultural homogeneity, which is considered important in IPA to explore culturally embedded meanings. Exclusion criteria included: (a) therapists with more than five years of CBT experience, (b) therapists without CBT training, (c) individuals with a current mental health diagnosis or undergoing treatment, and (d) those outside the specified age range.

### 2.3. Data Collection

Participants were identified through online professional platforms and forums, including Doctors Anytime, where their clinical experience and credentials are publicly documented. None of the participants were previously known to the researcher. Initial contact was made via email or telephone, inviting them to participate in the study. Following this, snowball sampling was employed, allowing participants to recommend other eligible novice CBT therapists, which helped to broaden the sample while maintaining relevance to the study’s focus. Interviews were scheduled at mutually convenient times. All interviews were conducted online via Teams during November 2024 and January 2025. Prior to participation, individuals received an information sheet and a consent form. After participation, they were provided with a debrief form reiterating their right to withdraw within two weeks post-interview. Data were collected through one-to-one, semi-structured interviews. This format offered a balance between structure and flexibility, enabling the researcher to guide discussions while allowing participants to share personal experiences freely. It also facilitated deeper exploration of responses and follow-up questions that could reveal insights (Ritchie & Lewis, 2003). An interview guide was designed with questions inviting participants to reflect on their personal and professional experiences (e.g., Can you describe some of the most significant challenges you have faced since you began practicing as a CBT therapist?), explore potential contributing factors (e.g., What personal or professional circumstances do you think might have contributed to these challenges?), and discuss their coping strategies and support systems (e.g., How do you typically cope with or manage these challenges?).

Prior to each interview, participants were reminded of the study’s purpose, the voluntary nature of their participation, and their right to withdraw at any stage without penalty. Interviews were audio-recorded with participants’ consent and stored securely on a password-protected memory stick, accessible only to the researchers. To preserve anonymity, participants were assigned unique codes derived from the first two letters of their surname and the last two digits of their phone number.

### 2.4. Data Analysis

Data were analyzed using Interpretative Phenomenological Analysis (IPA), a qualitative approach designed to explore in depth how individuals make sense of their lived experiences (Smith et al., 2009). IPA is particularly appropriate for this study because it allows a detailed examination of the professional and emotional journeys of novice CBT therapists, capturing both shared experiences and the nuances of individual meaning-making. All interviews were transcribed verbatim and analyzed manually, without the use of qualitative analysis software. This manual approach enabled the researchers to immerse themselves in the data, maintaining close engagement with the language, tone, and context of participants’ accounts. Both researchers independently coded the transcripts to enhance analytical rigor. Each transcript was read multiple times, with the researchers highlighting significant statements, phrases, and reflections that appeared to capture the essence of participants’ experiences. The next step involved developing initial codes, which consisted of descriptive, linguistic, and conceptual interpretations of the participants’ statements. These codes were not pre-determined but emerged inductively from the data, reflecting the idiographic focus of IPA. Following this, related codes were examined collectively to identify patterns and connections, forming provisional themes that represented broader aspects of the participants’ experiences. Throughout the analysis, the researchers engaged in iterative discussion and reflexive consideration. Analytical decisions, emerging interpretations, and thematic structures were regularly reviewed and debated until consensus was reached. This process ensured that the final themes reflected both the participants’ own perspectives and the interpretative insights of the researchers, in line with IPA’s double hermeneutic principle, whereby the researcher makes sense of the participant making sense of their experience. The iterative nature of IPA allowed for continual refinement of themes. This involved comparing individual accounts to identify convergences and divergences, considering how each participant uniquely navigated professional identity, emotional challenges, and coping strategies, while also capturing common patterns across the group. The final thematic structure thus represents a careful balance between idiographic attention to individual experiences and interpretative abstraction to generate meaningful patterns, providing a rich and nuanced understanding of the professional development journeys of novice CBT therapists.

### 2.5. Ethical Considerations

The study adhered to the University’s Good Scientific Practice guidelines and the Data Protection Act. Participation was voluntary, with informed consent obtained prior to data collection. Anonymity and confidentiality were maintained throughout. Participants were informed of their right to withdraw without penalty at any point, including up to two weeks post-interview, and to request removal of their data. They were also provided with details of mental health support services should any distress arise from their participation.

## 3. Results

The analysis of the interviews with novice CBT therapists yielded two main themes, each encompassing sub-themes. These themes capture participants’ experiences and strategies for managing the emotional and professional demands of their work, as well as their ongoing personal and professional development (Table 2).

### 3.1. Theme One: Professional Identity Challenges and Self-Beliefs

#### 3.1.1. From Theory to Practice and Fear of Failure

A major theme emerging from the interviews was the challenge of forming a professional identity while managing low self-confidence. Novice CBT therapists frequently reported intense self-questioning, doubt, and uncertainty when transitioning from theoretical knowledge to clinical practice. Many participants expressed fears of inadequacy, often questioning whether their skills and interventions were effective. GS987 reflected:

*“I think the biggest challenge has to do with the fact that as humans, when we start something new… this lack of experience can lead to a feeling of insecurity. That is, am I enough or not? Will I be able to truly support and help the person who comes to me? And many times, these thoughts come into conflict and thus create more uncomfortable feelings and insecurities, which at first work overwhelmingly.”*

Similarly, participant Ik09 described self-doubting thoughts related to professional competence:

*“One part has to do with my anxiety, with the feeling of adequacy, that is, am I adequate to be a CBT therapist or generally a CBT therapist at the first level? Am I doing this job? Can I handle the cases that have been assigned to me? Am I finally helping my clients?”*

These reflections highlight the difficulty early-career therapists experience in internalizing the role of an effective practitioner, linking professional identity closely to perceived self-efficacy. The struggle to attribute client progress to themselves further illustrates this challenge. Participant EP010 noted:

*“I see change, but I can’t believe it—I’m putting myself down, underestimating my role… even when progress is visible, I find it difficult to acknowledge my contribution.”*

A further challenge involved being perceived as inexperienced or underestimated due to youth. TK109 shared:

*“I feel that because I look younger than I am… as if in some way this is a manifestation of underestimation towards me. Which creates strange feelings for me, generally makes me feel uncomfortable, this immediate reaction to being comfortable.”*

Even when acknowledging that such perceptions might be self-imposed, these experiences influenced participants’ sense of professional identity and authority within sessions. Maintaining the flow of therapy sessions also emerged as a significant challenge. TK109 described:

*“To see if what I have understood reflects what she said… either in this way to give her the go-ahead to continue, or to make a summary so that I can formulate a question to move forward… Somehow, I deal with my anxiety that, for example, he just said something and I don’t know how to continue it, or I don’t know what to ask next.”*

These narratives illustrate how pauses and summaries serve as practical tools to reduce anxiety and maintain session structure. Early-career therapists additionally reported difficulty balancing client expectations for immediate progress with adherence to therapeutic protocols, creating internal dilemmas. SIN234 participant explained:

*“Many have a tendency to get well quickly, which is also difficult, and you need to not interfere with their logic but to explain to them how the therapeutic protocols work… little by little we get used to it.”*

#### 3.1.2. Emotional Involvement and Boundary Management

Managing emotional involvement while maintaining professional boundaries emerged as another significant theme. Participants reported intense emotional reactions during sessions, including sadness, grief, and vulnerability, which they felt compelled to suppress. GS987 shared:

*“In the self-injury situations I forced myself… I did not react, I just waited… because I felt bad interrupting the person who was describing this to me.”*

Similarly, EK101 described challenges in grief-related sessions:

*“It wouldn’t be good to show at that moment that I am emotional… I had to suppress my feelings to remain supportive.”*

Early-career therapists also struggled with setting boundaries, particularly regarding time, personal disclosures, and relational closeness. VK567 reflected on difficulties with time management:

*“It was very difficult to change this behavior until I couldn’t do anything else at the weekend or enjoy an excursion… answering every client message led to emotional strain.”*

Boundary challenges were also evident when clients displayed excessive friendliness or idealization. TK109 noted:

*“Something that concerns me in relation to the boundaries on the part of my client is the considerable friendliness and relaxedness she shows… as if she came to have a coffee with a friend.”*

GS987 described the risks of client idealization:

*“Some clients bring into the room an image of the therapist as a ‘lifeline’ or ‘God’… this kind of pressure can lead to disappointment and anger and potentially break down the therapeutic relationship.”*

#### 3.1.3. Managing Difficult Clinical Cases

Participants highlighted the challenge of handling emotionally intense or complex cases, including grief, self-harm, and personality disorders. GS987 shared:

*“A very strong emotional response was triggered during a case involving loss, such as the death of a child… it was intense and difficult to maintain my professional role.”*

Similarly, VK567 described self-harm cases as particularly challenging:

*“Whenever there is a description of a self-harm episode or an attempt, it triggers more intensity in me compared to other cases… professional detachment is difficult.”*

Personality disorders also elicited fear and self-doubt. SIN234 expressed apprehension regarding narcissistic clients:

*“I think that would scare me… they will underestimate you a lot… it would touch on my insecurities and be a little difficult for me.”*

VK987 and EK101 further highlighted the complexities of borderline and other personality disorders, emphasizing the difficulty of modifying deeply ingrained patterns through CBT or schema therapy. Collectively, these narratives illustrate the complex interplay of professional identity, self-efficacy, emotional regulation, and boundary management in novice CBT therapists, highlighting the multifaceted challenges inherent in early clinical practice.

### 3.2. Theme Two: Strategies for Emotional Regulation and Ongoing Professional Development

#### 3.2.1. Strategies for Emotional Unloading

Novice CBT therapists frequently encounter intense clinical situations that evoke strong emotional responses, often leaving them feeling overwhelmed or emotionally charged. Participants reported employing various personal strategies to decompress and restore emotional balance, highlighting the importance of both formal and informal support systems. Supervision and personal therapy emerged as critical tools for managing emotional strain. GS987 emphasized the role of personal therapy and supervision as essential outlets to: “…*decompress all these emotions we experience*.” Similarly, SIN345 highlighted the importance of supervision at the beginning of a CBT therapist’s career, framing it not only as a source of clinical guidance but also as a mechanism for reassurance:

*“I gain a bit of confirmation… it helps me feel not so inexperienced.”*

VK567 further echoed this perspective, noting that supervision provides critical support when cases become overwhelming:

*“…I feel that certain cases overwhelm me too much and I can’t manage them well.”*

Beyond formal supervision, participants emphasized the value of social support from colleagues, friends, and family. TK109 described talking with friends as a way to disconnect from work-related stress, stating that these conversations help her *“forget and enjoy herself”*, suggesting that temporarily stepping away from professional identity is vital for maintaining emotional balance. Similarly, IK09 described interpersonal interactions as:

*“…one of the most therapeutic and functional strategies for emotional relief… [my social environment] plays the most significant role”*

Participants also highlighted the importance of self-care activities, including personal time, journaling, physical activity, and reading. GS987 stressed the necessity of attending to personal needs, explaining:

*“…if our own cup is empty, we will not be able to fill the emotional and psychological cup of other people.”*

Journaling was repeatedly cited as a structured method for emotional processing and self-monitoring. TK109 shared that she records reflections of gratitude in her journal, while GS987 uses journaling to process feelings of “*discomfort and pressure*” following sessions. EK101 described journaling as mirroring client exercises:

*“…what we give them to do, I also do for myself… during particularly difficult weeks, when I felt drowned, I would engage in weekly reflections to identify what might have gone wrong.”*

Physical activity was also highlighted as an effective outlet. TK109 noted that exercise allows her to “*shift from mental fatigue to physical fatigue*” while EK101 described walking as a way to maintain emotional balance. IK09 framed physical activity as a deliberate form of distraction:

*“…engaging in something completely different… exercise helps a lot… so that the anxiety I feel, especially with new cases, isn’t as intense.”*

Finally, informal leisure activities such as reading were also considered central to emotional unloading. SIN234 reflected “*reading books and listening to music… helps me feel like I am gaining something,*”, emphasizing how these activities contribute to both emotional relief and personal growth. EK101 similarly noted that reading provides a mental escape and helps restore composure.

#### 3.2.2. The Need for Continuous Development

Participants also reflected on the personal growth and professional development fostered by their work as CBT therapists. Many noted enhanced self-awareness, empathy, and relational understanding as key areas of personal evolution. VK567, for example, described learning to “*fully accept the other person*”. highlighting the emotional satisfaction derived from cultivating this skill. EK101 similarly described the development of cognitive flexibility:

*“…working with clients helped me evolve my thinking and see things from many different angles… made me feel much more relaxed.”*

Participants reported enhanced empathy and perspective-taking abilities. IK09 noted that his work allowed him to “*think that this person is not what I see at that moment*”, demonstrating an ability to perceive beyond surface-level behaviors. EK101 reflected on the differentiation between personal and professional roles:

*“…I feel like two completely different persons… inside the therapy office, I adopt the professional role… outside, my reactions are different and more spontaneous.”*

This distinction illustrates the ongoing process of professional identity development and the importance of boundary-setting for emotional resilience. VK567 similarly noted that exposure to CBT tools improved her emotional regulation in personal life, allowing her to handle challenges with less intensity:

*“…challenges do not come with the same volume.”*

Participants expressed a strong desire for continuous professional growth. VK567 highlighted the importance of lifelong learning and expanding therapeutic approaches, particularly in mindfulness, person-centered, and compassion-focused therapies:

*“…knowledge is constantly advancing… CBT doesn’t stop; it’s a very large umbrella and has many branches.”*

EK echoed this commitment, emphasizing the complexity of CBT and the necessity of ongoing study. GS987 underscored professional development as an ethical responsibility, stating:

*“…we have to study, we have to make an effort, constantly… especially lately, I’m doing training in psychoeducation… cultivating more skills—in communication, in developing empathy towards our clients. This is extremely important! For us therapists.”*

Collectively, these narratives reveal that novice CBT therapists actively engage in strategies for emotional regulation while simultaneously pursuing continuous professional and personal development. The interplay of self-care, supervision, social support, and lifelong learning reflects a holistic approach to sustaining professional efficacy and psychological well-being.

## 4. Discussion

The present study explored the lived experiences of novice CBT therapists, focusing on the professional and emotional challenges they encounter, alongside the strategies they employ for emotional regulation and ongoing development. Two overarching themes were identified: (1) professional identity challenges and self-beliefs, and (2) strategies for emotional regulation and continuous growth. While previous studies have examined similar phenomena, this study contributes by detailing the experiences of Greek novice CBT therapists, highlighting context-specific challenges and coping strategies that have not been fully documented. These findings contribute to a growing body of literature on the early stages of psychotherapy practice, highlighting the complex interplay between competence, emotional resilience, and professional development in shaping the identity of early-career practitioners.

### 4.1. Development Professional Identity Formation and Self-Beliefs

Consistent with previous research, our findings underscore the centrality of professional identity formation in the early stages of a therapist’s career (Choudhury et al., 2019; Råbu et al., 2016). Participants frequently described self-doubt and anxiety when moving from theoretical learning to applied practice, echoing prior work showing that novice therapists often struggle to internalize their role as effective practitioners (Thériault et al., 2009). Perceived inexperience, compounded by external or self-imposed underestimation due to age or appearance, appeared to intensify feelings of inadequacy. These experiences align with evidence that lack of confidence in clinical skills can amplify perfectionistic tendencies, which in turn heighten stress and vulnerability to burnout (D’Souza et al., 2011). Importantly, our study adds to this literature by illustrating how these self-doubts manifest in real-time therapy sessions, providing concrete examples of moments where professional identity is challenged and strategies are applied to cope.

Participants’ difficulty in acknowledging their role in client progress reflects a potential mismatch between observable therapeutic outcomes and therapists’ internalized self-efficacy. This highlights a novel contribution: even when external outcomes are positive, novice therapists may fail to integrate these experiences into their professional self-concept, suggesting a target for training and supervision interventions. Moreover, the need to balance client expectations for rapid progress with adherence to structured protocols reflects the ethical and relational complexity of therapeutic work at this stage of development. By documenting these micro-processes, this study provides actionable insights for early-career therapist training, pinpointing where novices struggle with presence, flexibility, and decision-making.

The results also revealed challenges in maintaining session flow and making in-the-moment decisions, highlighting a developmental gap in clinical spontaneity. This resonates with Råbu et al.’s (2016) observation that novice therapists may experience a disruption in their sense of competence when their flexibility is tested, particularly in emotionally charged situations. Such difficulties may also reflect the limited skills and self-awareness characteristic of early professional development. For example, research has shown that novice counsellors often struggle to tolerate silence in sessions, as their attention becomes preoccupied with their own internal dialogue rather than remaining fully attuned to the client (Tran & Henriksen, 2015). This tendency to be “inside one’s head” has been linked to an underdeveloped capacity for presence and self-understanding, which can undermine therapeutic responsiveness. Furthermore, while many trainees are motivated by altruistic reasons for entering the profession (Beatty, 2012), others may be less conscious of underlying personal motives, including the wish to address their own unresolved needs (Kottler & Shepard, 2015). These hidden dynamics may compound the novice’s difficulty in engaging wholly with the client, thereby contributing to the challenges observed in this study around sustaining therapeutic flow and spontaneity. By highlighting these nuanced interactions between personal motives, self-doubt, and clinical practice, this study clarifies mechanisms through which novice therapists’ professional identities develop, offering concrete targets for training and supervision.

### 4.2. Emotional Involvement, Boundaries, and Clinical Complexity

The emotional demands of therapy, particularly in cases involving grief, self-harm, and personality disorders, were described as especially taxing. Participants’ accounts mirror previous findings that therapists, particularly those in the early stages of practice, are highly susceptible to intense emotional reactions when confronted with high-risk or high-intensity presentations (Råbu et al., 2016). The fact that these topics emerged as the most challenging may reflect several interrelated dynamics. First, such cases often fall at the limits of a novice therapist’s clinical repertoire, demanding advanced skills in containment, risk assessment, and emotional regulation that are still underdeveloped in the early stages of practice. Second, these clinical contexts strike at the heart of the therapist’s professional identity, amplifying fears of inadequacy and responsibility for client safety. This is consistent with earlier research linking exposure to emotionally charged material with precursors to burnout, including psychological strain, heightened self-doubt, and a sense of personal insufficiency (Demirtzidou & Tragantzopoulou, 2025; Tragantzopoulou et al., 2024). Finally, the centrality of these themes highlights the novice’s struggle to balance therapeutic presence with the emotional weight of clients’ narratives. Taken together, our participants’ difficulties suggest that early-career therapists may be at heightened risk of cumulative emotional fatigue, particularly in the absence of robust supervisory and organizational frameworks that scaffold their work with high-risk clients.

Boundary management emerged as another critical issue. Therapists described difficulties in maintaining time boundaries, responding to client communications outside of sessions, and navigating client idealization. Such challenges are well-documented in the literature, where they are associated not only with heightened emotional load but also with role confusion and ethical complexity (Skovholt & Rønnestad, 2003). Our findings suggest that these difficulties may be partly rooted in the novice therapist’s strong desire to help, which, although often motivated by altruistic intentions, can inadvertently blur professional boundaries. Therefore, our findings extend existing literature by showing how these boundary challenges interact with personal traits, altruistic motives, and digital communication pressures, providing a more granular understanding of why novice therapists struggle in these areas. As Zielinska (2015) notes, when early-career counsellors assume an excessive sense of responsibility for client outcomes, they may overextend themselves beyond the therapeutic frame—responding to messages, engaging outside session time, or striving to solve problems that exceed their role. The proliferation of digital communication technologies further complicates this terrain, as constant accessibility via calls, emails, or texts can create pressure to remain available and responsive, sometimes leading to what has been termed “boundary crossings” (Kottler & Shepard, 2015). For novice therapists, who are still consolidating their professional role, these dynamics can intensify self-doubt and contribute to emotional exhaustion.

### 4.3. Strategies for Emotional Regulation and Growth

Participants employed a variety of strategies for managing the emotional demands of their work, including supervision, personal therapy, social support, and self-care activities. These findings corroborate prior research showing that structured professional support, particularly clinical supervision, is central to mitigating distress and fostering resilience in novice therapists (Demirtzidou & Tragantzopoulou, 2025). Supervision was described not only as a space for case consultation but also as a resource for reassurance and validation, helping therapists counteract feelings of inexperience and self-doubt. This aligns with quantitative findings showing that therapist competence, rather than strict adherence to CBT techniques, predicts client improvement in anxiety treatment settings (Brown et al., 2013). Moreover, competence has been shown to decrease over time in the absence of ongoing supervision and training, highlighting the need for booster sessions and structured professional development to sustain therapist efficacy (Brown et al., 2013).

Social support from peers, friends, and family was also emphasized, aligning with studies highlighting the buffering effect of supportive relationships in reducing emotional strain (Thériault et al., 2009; Tragantzopoulou et al., 2024). Informal self-care practices such as journaling, physical activity, and reading served as additional outlets for emotional processing and restoration. Notably, some participants mirrored therapeutic tools in their own self-reflection (e.g., journaling gratitude lists), suggesting that clinical skills can cross-pollinate into personal resilience strategies. This observation resonates with recent findings emphasizing the importance of self-awareness and deliberate practice in therapist development. In a qualitative study of CBT trainees, self-awareness was found to emerge primarily in recognizing one’s skills and emotional experiences, whereas awareness of biases, values, and emotional regulation appeared less frequently and proved more resistant to change. These findings highlight that intentional reflective activities, such as reviewing therapy recordings and attending to one’s internal reactions, can enhance therapists’ insight into their own practice and promote greater professional growth (Pereira et al., 2024). Complementing these qualitative findings, research on 44 graduate psychology students treating simulated patients found that personal traits such as resilience and extraversion modestly predicted initial clinical micro-skills, though overall skill variation was limited. This highlights the importance of fostering personal qualities alongside structured CBT training to enhance novice therapists’ competence, while underscoring the need for further research with larger samples to fully understand skill development (Schaffrath et al., 2024).

The commitment to continuous professional development was another strong feature of participants’ narratives, reinforcing prior claims that lifelong learning is a hallmark of sustainable therapy practice (Skovholt & Rønnestad, 2003). For many, expanding therapeutic knowledge was not only a professional necessity but also an ethical responsibility, reflecting a proactive orientation toward skill enhancement and adaptability.

### 4.4. Strengths and Limitations

This study provides valuable insights into the lived experiences of novice CBT therapists by foregrounding their voices and exploring both professional and emotional challenges. The qualitative design and thematic analysis enabled the capture of nuanced perspectives, shedding light on issues that may remain hidden in quantitative approaches. The focus on early-career therapists also contributes to a deeper understanding of a critical but underexplored phase of professional development. A key strength of this study is its contextual specificity. By focusing on Greek CBT therapists, it highlights culturally and systemically relevant experiences that may differ from other populations, providing insights for cross-cultural comparison and adaptation of training programs.

Nonetheless, several limitations should be acknowledged. The sample was relatively small, though this is consistent with IPA methodological guidelines, which emphasize depth over breadth of analysis. All participants were Greek CBT therapists, and thus the findings may not fully reflect the experiences of novice practitioners in different cultural or training contexts.

### 4.5. Implications for Training and Practice

The study contributes actionable guidance for training programs by specifying which aspects of professional identity, boundary management, and emotional regulation require targeted support, thereby moving beyond general recommendations. Training curricula should more explicitly address the developmental nature of professional identity, helping novice therapists to contextualize self-doubt as a normal stage of growth rather than a marker of incompetence. Structured opportunities for reflective practice, including facilitated discussions on self-beliefs and therapeutic presence, could foster greater self-awareness and resilience.

Evidence suggests that training in CBT requires more than theoretical instruction alone. Rakovshik and McManus (2010) showed that while a minimum level of theoretical input is necessary, it is insufficient to achieve behavioral change without additional formative feedback, such as continuous supervision. Multicomponent training approaches—combining workshops, supervised practice, and active learning strategies—have been shown to be more effective than single-method approaches such as reading or stand-alone workshops (Beidas & Kendall, 2010; Herschell et al., 2010; Frank et al., 2020). Systematic reviews also highlight that extensive training hours (>137) produce stronger gains compared to brief training formats (Rakovshik & McManus, 2010), while instructor-led and blended online formats show moderate improvements, particularly when structured around specific skills (Henrich et al., 2023). These findings underline the importance of embedding sustained supervision and varied training modalities within professional development pathways.

Importantly, our study suggests that certain clinical presentations—particularly grief, self-harm, and personality disorders—pose distinctive challenges for novice CBT therapists. Training programs should therefore provide more structured preparation for working with these complex areas, including experiential learning, case simulations, and supervised exposure to high-risk or emotionally charged presentations. Emphasizing skills in emotional regulation, risk management, and containment strategies may strengthen therapists’ ability to navigate such cases without feeling overwhelmed. Bennett-Levy (2006) emphasizes that therapeutic competence requires not only technical and conceptual knowledge, but also interpersonal skills and complex reflective abilities, which can be cultivated through structured and supervised practice.

Boundary-setting also deserves particular attention, with dedicated training on managing professional limits, client communications outside of sessions, and the challenges introduced by digital technologies. Embedding practical scenarios and role-plays into training could better prepare therapists for these realities.

Supervision should be framed as a multidimensional resource that provides not only case guidance but also emotional support, validation, and modeling of professional boundaries. Supervisors can play a key role in helping therapists tolerate uncertainty, manage the emotional weight of complex cases, and integrate their professional identity with greater confidence. This finding aligns with empirical evidence demonstrating that structured models of CBT training and supervision can significantly improve both therapist competence and client outcomes (O’Keeffe et al., 2016). Reviews further confirm that additional supervision contributes directly to both therapist competence and improved client outcomes (Henrich et al., 2023; Frank et al., 2020).

Finally, training programs should encourage early-career therapists to engage in ongoing professional development as both a skills-enhancement and self-care strategy. Continuous professional training has been identified as central to long-term competence and professional identity development (Rønnestad et al., 2019). Structured workshops on self-care, emotional regulation, and reflective use of therapeutic tools may help mitigate the risk of cumulative emotional fatigue and burnout while supporting therapists’ growth across their careers.

## 5. Conclusions

This study underscores the complex interplay between professional identity, emotional resilience, and skill development in novice CBT therapists. Participants’ accounts reveal the unique vulnerabilities of early-career practice, including heightened self-doubt, boundary challenges, and emotional strain when working with complex cases. At the same time, the use of supervision, peer support, and continuous learning reflects a proactive engagement with growth and resilience. By integrating these insights into training and supervision structures, the profession can better support novice therapists in navigating the transition from student to practitioner, laying the foundation for sustainable and competent clinical practice.

## Figures and Tables

**Table 1:** Participants’ Characteristics.

Pseudonym	Age	Gender	Years of Practice
SIN234	23	FEMALE	3 YEARS
IK09	29	MALE	2 YEARS
GS987	27	FEMALE	5 YEARS
VK567	24	FEMALE	4 YEARS
EK101	24	FEMALE	4 YEARS
EP010	25	FEMALE	4 YEARS
TK109	27	FEMALE	1 YEAR

**Table 2:** Themes and Sub-Themes.

Main Theme	Sub-Themes	Description
**Theme 1: Professional Identity Challenges and Self-Beliefs**	1.1. From Theory to Practice and Fear of Failure	Challenges in transitioning from theoretical knowledge to clinical practice, self-doubt, and fear of inadequacy.
	1.2. Emotional Involvement and Boundary Management	Struggles with intense emotional reactions, setting boundaries, and managing relational closeness with clients.
	1.3. Managing Difficult Clinical Cases	Handling complex or emotionally charged cases, including grief, self-harm, and personality disorders.
**Theme 2: Strategies for Emotional Regulation and Ongoing Professional Development**	2.1. Strategies for Emotional Unloading	Use of supervision, personal therapy, social support, self-care, journaling, physical activity, and leisure to manage emotional strain.
	2.2. The Need for Continuous Development	Ongoing personal and professional growth, enhancing self-awareness, empathy, emotional regulation, and commitment to lifelong learning.

## Data Availability

Data can be made available upon reasonable request.

## Abbreviations

The following abbreviations are used in this manuscript:

CBT	Cognitive Behavioral Therapy
OCD	Obsessive-compulsive Disorder
PTSD	Post-traumatic Stress Disorder
MBCT	Mindfulness-Based Cognitive Therapy
DBT	Dialectical Behavior Therapy
IPA	Interpretative Phenomenological Analysis
